# Is There an Interplay between Oral Microbiome, Head and Neck Carcinoma and Radiation-Induced Oral Mucositis?

**DOI:** 10.3390/cancers13235902

**Published:** 2021-11-24

**Authors:** Patryk Gugnacki, Ewa Sierko

**Affiliations:** Department of Oncology, Medical University of Bialystok, 15-025 Bialystok, Poland; onkologia@umb.edu.pl

**Keywords:** microbiome, head and neck cancers, oncogenesis, radiation-induced oral mucositis, radiotherapy

## Abstract

**Simple Summary:**

Human microbiome research is rapidly expanding, including a variety of clinical medicine and public health disciplines. Commensal microbiome has a significant impact on shaping homeostasis of the organism, as well as the development of pathological states. A number of studies indicate that changes in the human microbiome could determine an oncogenic effect by, among other things, inducing chronic inflammatory response, instigating cellular antiapoptotic signals or modulation of anticancer immunity. The presence of pathogenic species also contributes to the exacerbation of the complications of the treatment applied, such as radiation-induced oral mucositis, which is the most common side effect of the treatment. A better understanding of the interplay between oral microbiome and head and neck cancers might provide an important step towards a more effective treatment for this highly malignant tumor. The following review is a complex revision of the up-to-date research on the oral microbiome and its impact on the development of head and neck cancer and radiation-induced oral mucositis.

**Abstract:**

Head and neck carcinoma is one of the most common human malignancy types and it ranks as the sixth most common cancer worldwide. Nowadays, a great potential of microbiome research is observed in oncology—investigating the effect of oral microbiome in oncogenesis, occurrence of treatment side effects and response to anticancer therapies. The microbiome is a unique collection of microorganisms and their genetic material, interactions and products residing within the mucous membranes. The aim of this paper is to summarize current research on the oral microbiome and its impact on the development of head and neck cancer and radiation-induced oral mucositis. Human microbiome might determine an oncogenic effect by, among other things, inducing chronic inflammatory response, instigating cellular antiapoptotic signals, modulation of anticancer immunity or influencing xenobiotic metabolism. Influence of oral microbiome on radiation-induced oral mucositis is expressed by the production of additional inflammatory cytokines and facilitates progression and aggravation of mucositis. Exacerbated acute radiation reaction and bacterial superinfections lead to the deterioration of the patient’s condition and worsening of the quality of life. Simultaneously, positive effects of probiotics on the course of radiation-induced oral mucositis have been observed. Understanding the impact on the emerging acute radiation reaction on the composition of the microflora can be helpful in developing a multifactorial model to forecast the course of radiation-induced oral mucositis. Investigating these processes will allow us to create optimized and personalized preventive measures and treatment aimed at their formation mechanism. Further studies are needed to better establish the structure of the oral microbiome as well as the dynamics of its changes before and after therapy. It will help to expand the understanding of the biological function of commensal and pathogenic oral microbiota in HNC carcinogenesis and the development of radiation-induced oral mucositis.

## 1. Introduction

Head and neck cancer (HNC) constitutes a heterogeneous group of tumors including, among others, malignant neoplasms of lips, oral cavity, pharynx (usually divided into naso-, oro- and laryngopharynx), larynx, paranasal sinuses, or salivary glands. HNC is one of the most common human malignancy types and it ranks as the sixth most common cancer. Each year, nearly 900,000 people worldwide are diagnosed with HNC and more than half of them die (approximately 450,000 per year) [1]. A common trait for these tumors is their biology—the vast majority (over 90%) are histologically diagnosed as squamous cell carcinomas [2]. Therefore, the term used to describe this type of tumor is head and neck squamous cell carcinoma (HNSCC). Despite significant progress in early detection and treatment, HNC is still a serious challenge in clinical practice.

The incidence rates and location of HNC vary worldwide, which is mainly related to different lifestyles [1,3]. The main risk factors include tobacco use and alcohol consumption. The prevalence of HNC is higher in men than in women (5.8 and 2.3 per 100,000 individuals, respectively) [4]. Currently, there is an increase in the incidence rate among women. The suspected reason for this phenomenon is the increase in tobacco use among women when compared to previous eras [5]. Asia has the highest number of new cases [1]. It is worth mentioning that the eating habits and associated risk factors of HNC in the Asian population differ significantly from those of people on other continents—the use of betel quid, with or without tobacco, is more frequent than alcohol consumption [1,3].

## 2. HNC—Risk Factors and Carcinogenesis

*Human Papillomavirus Virus* (HPV) plays an important role in HNSCC carcinogenesis [6]. It is a double-stranded deoxyribonucleic acid (DNA) virus, highly tissue-specific and infects both the cutaneous and mucosal epithelia [7]. The virus is transmitted mainly by sexual activity, including oral sex, but also skin-to-skin contact [7]. Currently, more than 220 HPV genotypes have been described. Mainly types 16 (over 70% of all HPV-positive cases) and 18 (over 14% of all HPV-positive cases) show a high oncogenic potential, but also 19 other types have been associated with an increased incidence of this type of cancer [8]. Beyond HNSCC, the involvement of HPV squamous cell carcinogenesis has been demonstrated in other locations, such as the uterine cervix, the vagina, the vulva, the penis, the anus and the rectum [9]. Specific affinity of HPV carcinogenesis has been established for oropharyngeal squamous cell carcinoma (OPSCC). The percentage of HPV-positive OPSCC in the United States is approximately 70% [6]. However, it is still debatable whether HPV plays a significant role in other HNSCC sub-sites, such as oral cavity cancers [10]. Chaturvedi et al. [6] estimated that by 2020, the frequency of HPV-positive OPSCC will be more prominent than the rate of HPV-related cervical cancer. Presumably half of all HNC will be related to HPV by 2030. It has been observed that patients with HPV-positive HNC are on average 3–5 years younger, often smoking less frequently, and do not consume high-percentage alcohol [11].

Due to the development of HPV vaccines, it has become possible to reduce the number of cervical cancer cases [12]. There are currently three types of vaccines licensed: quadrivalent vaccine, Gardasil (Merck; targeting HPV type 6, 11, 16, 18); bivalent vaccine, Cervarix (GlaxoSmithKline, targeting HPV type 16, 18); and HPV vaccine, Gardasil 9 (Merck; targeting HPV type 6, 11, 16, 18, 31, 33, 45, 52, 58), which reduce the risk of developing cervical cancer respectively by 70%, 84% and approximately 90% [12]. It is suspected that HPV screening and the use of vaccinations should, in addition, reduce the risk of HPV-positive HNC, however, at the moment there are insufficient data to support this relationship [13]. Nevertheless, it has been proved that a HPV vaccine prevents both oral and oropharyngeal HPV infections, with a significant reduction in prevalence, up to 90%, among young adults [14,15,16,17]. Further longitudinal studies, cost-efficiency analysis and risk-benefit analysis are necessary to assess the influence of HPV vaccinees on the incidence of HNC.

Virus-induced carcinogenesis is associated with the influence of HPV on the cell cycle. The HPV genome contains six non-structural genes (early genes: *E1, E2, E4, E5, E6,* and *E7*) and two structural genes (late genes: *L1* and *L2*) [18]. The *E5, E6* and *E7* genes are directly related to HPV-dependent neoplastic transformation. Integration of viral genetic material with the host genome leads to synthesis of proteins encoded by the *E6* and *E7* genes, which are direct oncogenes and lead to cell cycle regulation disorders [8]. Protein E6 binds to the suppressor protein p53 via E6AP (ubiquitin-protein ligase E3A), which consequently leads to proteasomal degradation [19]. On the other hand, the E7 protein causes the cell to move into the S phase of the cell cycle by binding to the pRb protein (retinoblastoma protein) and dissociates it from transcription factor E2F. This sequence of events leads to the loss of its suppressor functions [20]. The resulting E5 protein and the epidermal growth factor receptor (EGFR) complex leads to the constant activation and proliferation of cells [21]. The following changes, which disrupt the activity of proteins regulating the cell cycle, lead to uncontrolled cell proliferation and cancer development. 

It is of note that tobacco use has been proposed to influence HPV-positive HNSCC progression. Cigarette smoke decreases expression patterns of micro-ribonucleic acid (miRNA) in epithelial cells (mainly *miRNA-133a-3p*) responsible for inhibiting proliferation of HNC cells. Down-regulation of *miRNA-133a-3p* is related to an increased EGFR expression level, which contributes to tumor growth [22,23].

Poor oral hygiene can also be considered as an independent risk factor for HNSCC, especially oral cavity cancers. The factors contributing to poor oral hygiene include, among others, irregular toothbrushing habits, missing teeth, malnutrition, poor socioeconomic status, low levels of education, tobacco, alcohol consumption and fewer dental visits. A lack of oral hygiene may be the reason for even a 12-fold increase in the risk of contracting HNSCC. It is also associated with poorer survival rates for HNC patients [24]. Farquhar et al. [25] revealed, based on a study of 1381 HNC-patients, that having >10 dental visits in the previous 10 years was linked to a lower risk of death. In addition, Chang et al. [24] observed that a lack of frequent dental check-ups and general poor oral hygiene were also linked to a lower HNC patient survival rate. Table 1, Table 2, Table 3 and Table 4 summarize the studies examining the impact of oral risk factors on the development of HNC.

Inadequate oral hygiene can facilitate the growth of photogenic bacteria in the oral cavity. Dysbiosis leads to local and systemic inflammation, which is known to promote oncogenesis; an example of this is *Helicobacter Pylori* infection in the stomach and the development of mucosa-associated lymphoid tissue lymphoma (MALT lymphoma) [26]. One of the most commonly known mechanisms is overexpression of Toll-like receptor 5 (TLR5). TLR5, as a pattern recognition receptor, binds flagellin, a crucial globulin bacterial protein. This initiates the canonical proinflammatory pathway by recruitment of the nuclear factor—kappa B (NF-κB) and production of proinflammatory cytokines, such as interleukin-8 (IL-8) or tumor necrosis factor *(*TNF) [27].

*Fusobacterium nucleatum*, one of the prominent players in periodontal disease, can also affect the development of HNSCC by promoting invasion of squamous cell carcinoma through association with epithelial mesenchymal transition. *Fusobacterium nucleatum* causes overexpression of partial epithelial–mesenchymal transition-related genes (p-EMT) such as *SERPINE1, ITGA5, TGFBI, P4HA2, CDH13*, and *LAMC2* and reduced expression of epithelial markers such as E-cadherin. The occurring changes, such as loss of cell polarity and cell–cell adhesion, lead to an increase of invasiveness of cancer cells manifested by invasion through basement membrane and increase in the risk of metastasis [28].

## 3. Management and Prognosis of HNSCC Patients

Management of patients with head and neck cancer depends on the location of the malignant neoplasm, the stage of the disease at the time of diagnosis and the histological assessment. Proper staging is essential for selecting personalized anticancer treatment [48].

For early-stage oral cavity cancers (stage I-II), a treatment regimen includes surgical radical resections or radiotherapy, which show similar locoregional control and survival rates. However, they have not been compared in randomized trials [49]. In locally advanced disease, the cooperation of multidisciplinary teams is crucial for selecting the optimal personalized treatment option. The basis of the treatment is surgical resection supplemented with adjuvant radiotherapy (RT) or chemoradiotherapy (CRT),as well as CRT or RT combined with EGFR inhibitor—cetuximab. Primary systemic therapy and post-operative systemic therapy standards are mainly cisplatin-based regimens [50]. For unresectable, recurrent or metastatic diseases, preferred regimen includes treatment with an anti–programmed death-1 (PD-1) monoclonal antibody (nivolumab or pembrolizumab) or cisplatin or carboplatin/5-fluorouracil with cetuximab [51,52]. In cases of nasopharyngeal cancer (NPC), the treatment of choice includes definitive RT or concurrent CRT.

The 3–5-year overall survival (OS) in stages I-II HNC after radical treatment is 70–85%. In stage III-IV incidences of the disease, the OS is decreased to about 50% [53,54]. Of note is that HPV-positive oropharyngeal cancer patients have a better prognosis than non-HPV-associated HNC patients [8].

## 4. Radiotherapy-Induced Oral Mucositis

The most frequently used radiotherapy techniques in modern radiotherapy departments include Intensity-Modulated Radiation Therapy (IMRT) or Volumetric Modulated Arc Therapy (VMAT) with a total cumulative dose of 70 Gy (gray) by 2.0 Gy per day delivered over 7 weeks in definitive treatment and/or 60–66 Gy by 2.0 Gy over 6 to 6.5 weeks in adjuvant treatment [50]. In patients with HNSCC undergoing RT, radiation-induced inflammation of the oral and/or pharyngeal mucosa occurs in the irradiated area (RIOM, radiation-induced oral mucositis). It is associated with significant discomfort, pain, difficulty in eating, and loss of taste and appetite, which directly lead to weight loss and dehydration and significantly reduce patients’ Quality of Life (QoL). Exacerbated acute radiation reaction often leads to the need for hospitalization, antibiotic and antifungal therapy administration, or even the need for feeding tubes or gastrostomy. Patients with RIOM have a fourfold higher relative risk of septicaemia than individuals without mucositis [55]. Oral complications have a major impact on the cancer treatment—mucositis can entail the need for reducing the dose of chemotherapy and inexpedient breaks in RT, which would have a negative impact on overall treatment effectiveness and patients’ prognoses [56]. Another important factor related to RIOM is that mucositis leads to higher resource use, appointments, hospitalizations, and prolonged hospitalizations, resulting in a significant increase in the cost to the patient and health system across a variety of cancer therapies and diagnoses [57].

Despite the use of modern highly sophisticated RT techniques, such as IMRT or VMAT, which allow reduction of the radiation dose to healthy surrounding tissues, this complication still poses many problems in clinical practice. The severity of RIOM is influenced by radiation-related factors: fractional dose, total cumulative dose, total volume of irradiated tissues (e.g., lower incidence of RIOM RT delivered to hypopharyngeal carcinoma resultant from lower radiation dose deposited in the oral cavity [58]) and overall treatment time, use of adjuvant treatment as well as other, not fully understood individual factors that affect response to RT. According to Vera-Llonch et al. [58], RIOM is more likely to occur in HNC patients who receive cumulative radiation doses >50 Gy with concurrent chemotherapy. It is known that the risk factors for the development of severe RIOM include, among others, being male, oropharyngeal cancers, the presence of anaemia, leukopenia or lymphopenia, concomitant CRT and oral feeding. Genetic factors, such as drug metabolism, TNFα gene polymorphism or polymorphism of genes responsible for protection against reactive oxygen species (ROS), also affect the severity of RIOM [59].

Preventive factors, such as proper oral hygiene, good dental health and a lack of irritating factors in the form of, e.g., incorrect fillings or dentures, reduce the risk of RIOM development and the severity of this complication [59].

Oral mucositis is most common and severe in patients undergoing RT treatment for oral cavity and oropharyngeal cancers [60]. The incidence of RIOM reaches 100% if all stages of severity are taken into account. However, incidence varies depending on tumor location, radiation dose and schedule, and the use of concomitant chemotherapy [61]. An important observation is also that the incidence of complications of anticancer therapy is underestimated, which also applies to RIOM [62].

Severe oral mucositis (grade III/IV in European Organisation for Research and Treatment of Cancer/Radiation Therapy Oncology Group scale (EORTC/RTOG scale)) develops in 39% of all patients undergoing anticancer treatment and varies on RT method, reaching the highest value in the case of altered fractionation RT (up to 57%) [61]. Severe oral mucositis develops mostly, up to 80%, in the area of the so-called “boost” (area of increased RT dose) and rarely in the remaining volume of irradiated mucous membranes, where grade I/II constitutes a total of over 90% [63]. It has been stated that adding chemotherapy to radiotherapy is equal to an additional dose of radiation, which raises the risk of mucosal Grade 3 toxicity fourfold over radiation therapy alone [64].

## 5. Pathogenesis of RIOM

The purification of damaged tissue structures from the remnants of damaged cells and pathogenic microorganisms leads to the initiation of the inflammatory process [60,62,65]. In the final stages, inflammation also induces repair processes using both the anti-inflammatory activity of immune system cells and the regenerative potential of the population of stem and progenitor cells. The main goal of this stage is to rebuild the integrity of tissues, including the epithelium of the mouth and pharynx, which is a natural barrier against the external environment re-inhabited by the commensal flora of the oral cavity [65].

The pathogenesis of RIOM is multifactorial and is the result of subsequent biological events [62]. Five overlapping biological stages, proposed by Sonis [62], describe the pathogenesis of RIOM. They include: initiation, the primary damage response (messaging and signaling), amplification, ulceration, and healing [62,66]. 

The initiation stage of oral mucosa damage starts immediately after delivery of radiation dose (and/or chemotherapy) and includes DNA and non-DNA injury with production of reactive oxygen species (ROS) via ionization of intracellular water [67]. 

Radiation causes tissue damage due to direct and indirect mechanisms. Direct and indirect effects of radiation, when combined, initiate a cascade of biochemical and molecular signaling activities that can reverse the damage or result in irreversible physiological changes in the cell or its death. The direct effect of radiation is caused by interactions with target macromolecules, distraction of atomic structures and damage to DNA strands, which leads to apoptosis and reduction of basal epithelial stem cells, as well as cells in submucosa. Oxidative products of water radiolysis are the main mechanisms of indirect radiation injury [68]. Water absorbs energetic radiation, which causes excitations and ionizations, resulting in the formation of free radicals, which can then target other essential molecules. Oxidative stress, due to ROS oxidating properties, leads to cell death and organella damage, which causes the release of additional ROS from mitochondria. Radiation-induced oxidative stress has the potential to spread from targeted cells to non-targeted bystander cells through intercellular communication mechanisms. Bystander cells’ progenies often undergo oxidative metabolism disruptions and show a broad variety of oxidative damage, including protein carbonylation, lipid peroxidation, and increased rates of spontaneous gene mutation [68]. Reactive oxygen species are crucial mediators for consecutive biological events. In addition, due to cell damage, release of inflammatory transmitters, changes in secretion of salivary glands, and neutropenia play a part in RIOM pathogenesis as indirect mechanisms [59].

The next stage, primary damage response, is initiated as a consequence of changes induced in the first phase, and also by the release of endogenous molecular structures, such as DNA, RNA, and proteins, commonly known as endogenous damage-associated molecular pattern [69]. Radiation and ROS lead to the activation of at least 14 pathways that alternate biological control mechanism and are associated with the development of RIOM (see Table 5.) 

It is currently believed that the most important and one of best-known mechanisms is the NF-κB pathway. In normal cells, activation of NF-κB pathways leads to apoptosis due to upregulating maximum 200 genes (including proinflammatory cytokine genes, i.e., cyclooxygenase-2, interleukin-1B, interleukin-6, inducible NO-synthase, superoxide dismutase and adhesion factors) [70]. Additional proinflammatory factors exacerbate or prolong tissue injury by interaction with target molecules or activation of NF-κB in other cells. NF-κB activation also leads to the production of anti-apoptotic and pro-apoptotic factors (i.e., BCL-2 gene family (B-cell lymphoma 2)—BAX (Bcl-2-associated X protein), BCL-X1 (B-cell lymphoma/leukemia-x long)), which determines changes in normal mucosa tissue. Radiation can disturb balance and cause overexpression of pro-apoptotic BAX and put healthy cells on the programmed cell death pathway [67].

Simultaneously, radiation alters the ceramide and sphingomyelin pathway by activating sphingomyelinase and ceramide synthase (both acid and neutral) and hydrolyzing cell-membrane lipid sphingomyelin. Increased ceramide levels induce the process of apoptosis through CAPK kinases (ceramide-activated protein kinases), MAPK (mitogen-activated protein kinases), kinase cascade SAPK/JNK (stress-associated protein kinase/Jun N-terminal kinase) and CAPP (ceramide-activated protein phosphatase). Moreover, ceramides can activate the intrinsic pathway of apoptosis by altering inner mitochondrial membrane potential and release of cytochrome c [71].

Radiation also affects submucosal cells. Activation of activator protein 1 (AP1) leads to secretion of metalloproteinase (MMP), which damages collagenous subepithelial matrix and epithelium base membrane and potentially exacerbates injury and allows the promotion and dissemination of other pro-inflammatory and pro-apoptotic signals [67].

Signal amplification. Transcription factors induced in primary damage response can positively or negatively affect the local cellular response. Pro-inflammatory cytokines such as TNFα (tumor necrosing factor-alpha) not only lead to programmed cell death but also create a positive-feedback loop and amplify other processes that are part of RIOM [62,67]. The leading example is TNFα, whose influence is multifactorial. (i) TNFα is an activator of NF-kB and sphingomyelinase pathways. (ii) TNFα a, through another TNFα receptor family, activates MAPK and causes event cascade—activation of JNK and AP1, leading to increased production of MMP. (iii) TNF-a together with IL-1 stimulate the platelet-activating factor via inflammatory cells (e.g., neutrophils and macrophages) and trigger the arachidonic acid cascade, which encourages the growth of prostaglandin E2 and other proinflammatory mediators, resulting in more tissue injury. 

Event cascade and accumulation of biological changes initiated through radiation delivery leads to clinically relevant mucosa injury. It occurs as ulceration exceeding to submucosa—in many cases covered with pseudomembrane composed mainly of fibrinous excaudate. Due to mucosa damage, nerve endings are exposed, resulting in the occurrence of pain and loss of function. Simultaneously, ulceration is the portal of entry for microorganisms, such as bacteria or fungi, and it causes a higher relative risk of septicaemia. In animal studies, in the inflamed epithelium, the number of pathogenic bacteria is 300 times higher than in a healthy epithelium [67]. Bacterial colonization plays an essential role in the pathogenesis of RIOM. Lipopolysaccharides, lipoteichoic acid, cell wall antigens, and α-glucans released from the bacterial cell wall can stimulate further secretion of proinflammatory cytokines by macrophages located in submucosa. For instance, by interacting with the membrane receptor CD14 (cluster of differentiation 14), lipopolysaccharide (LPS) activates immune responses by inducing the development of cytokines such as TNFα, IL-1, and IL-6, which together lead to an increase in changes caused by radiation [72].

In most cases, RIOM heals spontaneously 4–12 weeks after anticancer treatment is completed. The healing stage of mucositis is perhaps the least recognized of all the stages [62]. Nowadays, it is believed that submucosal signaling promotes proliferation, migration and differentiation of the epithelium cells, in which the most important is the activation of intrinsic tyrosine kinase [66,67]. The structure of the reconstituted submucosa is not identical to that of the submucosa prior to mucotoxic disruption, even after the epithelium has been fully replenished [73].

## 6. RIOM Manifestation

Clinical manifestation of acute radiation-induced oral mucositis depends on its severity. Typically, RIOM occurs 2.5 weeks after the start of RT and lasts for 2–3 weeks after the procedure is completed. The labial, buccal, and soft palate mucosa, as well as the floor of the mouth and the ventral surface of the tongue are the most common sites [74]. Even though RT or CRT causes tissue damage immediately after deposition of radiation, the clinical presence of mucosa and submucosa during the primary damage stage is normal [62]. The first symptoms appear after 10–20 Gy dose is deposed—hyperkeratosis and erythema of oral mucosa occurs by the second week of treatment and is accompanied by pain and dysphagia. In the third week (over 20 Gy dose), the mucous lining begins to erode, and focal epithelial lesions form progressively. In the fourth week (over 30 Gy dose), previously formed lesions develop ulcerations, often covered with pseudomembrane composed of fibrin, keratin and death cells excaudate. Necrosis and intense bleeding develop infrequently, but may be observed in patients with CRT [75]. Late complications of RT develop 3 months after completion of RT treatment. The most frequently reported late oral effects include, among others, xerostomia, increased exposure to mucosal infections, pain, sensory disturbances, and dental caries [76]. Due to vascular and soft tissue damage, atrophy and mucosa vulnerability, there is a risk of chronic ulcers and/or osteoradionecrosis [75].

## 7. RIOM Assessment

Currently, there are various scales available to assess the level of RIOM intensity. Comparison of the scoring systems is presented in Table 6. Each of the developed questionnaires focuses on different factors. The World Health Organisation Oral Toxicity Scale (WHO scale) is the most commonly used. It assesses the severity of RIOM on a 4-grade scale, focusing on anatomical, symptomatic, and functional elements of oral mucositis. Other equally frequently used scales are RTOG/EORTC (Radiation Therapy Oncology Group/European Organisation for Research and Treatment of Cancer) and CTCAE v5.0 (Common Terminology Criteria for Adverse Events). Additionally, research-dedicated scales, such as OMAS (Oral Mucositis Assessment Score) and the more extensive OMI (Oral Mucositis Index) consist of 34 items, each on a scale from 0 to 3 (normal to severe), which provide highly quantitative outputs based on a set of parameters that are narrowly defined. Of interest is Dische’s scale, which combines the assessment of clinical symptoms such as erythema, epitheliolysis, matted mucosa, oedema, bleeding, and ulceration with clinical symptoms such as discomfort and difficulty in swallowing and mucosal pain [77].

## 8. Human Microbiome and Oral Microbiome

The human microbiome is defined as the collection of microorganisms and their genetic material and its products residing within the mucous membranes [85]. It is common knowledge that the individual composition of commensal microflora has a significant impact on the health and the development of inflammatory diseases [86]. Significant progress in understanding the impact of the microbiome on the processes of shaping homeostasis of the organism as well as the development of pathological conditions is possible thanks to the use of the latest advances in molecular biology, namely sequencing, including New Generation Sequencing (NGS). Application of the 16S sequencing technique ribosomal RNA (rRNA), combined with available bioinformatics tools, enables a detailed analysis of the species and quantitative composition of the commensal microflora in a much more precise way than in previously available methods [87].

The studies applying NGS allowed for the identification of over 600 bacterial species-level phylotypes. Dewhirst et al. [88] examined samples from healthy people and patients with caries, pulp diseases and periodontal diseases. This study allowed for the creation of the Human Oral Microbiome Database (HOMD; www.homd.org). According to HOMD, approximately 57% of oral species have been formally named, 13% have been cultivated but remain unnamed, and 30% are uncultivated. Complementary to this database is the Zaura et al. [89] study. They identified a healthy core microbiome containing 78 types of bacteria and 34 higher taxonomic units that all occurred in examined individuals who did not show the presence of oral diseases. It can be assumed that previously uncharacterized bacterial colonies play an important role in the pathogenesis of numerous oral diseases. 

The oral cavity differs from all other human microbial environments in that it contains both shedding (mucosa) and stable surfaces for microbial colonization (teeth or dentures). The special environment of the oral cavity, with a stable saliva pH of 6.5–7.0, moisture, and its average temperature of 37 °C creates favorable conditions necessary for the growth of microorganisms [90]. This inherent property of the oral cavity provides opportunities for a wide variety of microbiota [89]. In periodontal health, the oral microbiome represents a well-balanced dynamic ecosystem that generally tends to remain within its typical values, far from values typical of periodontitis [91]. 

In the oral cavity, most habitats were dominated by *Streptococcus*, but these were followed in abundance by *Haemophilus* in the buccal mucosa, *Actinomyces* in the supragingival plaque, and *Prevotella* in the immediately adjacent (but low oxygen) subgingival plaque [92]. However, dysbiosis, a breakdown of the microbial homeostasis, induces oral disease and increases the risk of systemic diseases [93].

Oral microbiome may show large and rapid changes in composition and activity, both spatially and temporally. It has been indicated that dynamic changes in the composition of the microbiome occur with changes in the host. These multiplex, nonequilibrium dynamics are the result of many host-dependent factors that can be classified into two different groups: intrinsic and extrinsic. Genetic factors such as polymorphism in miRNA202, involved in hBD1 (human ß-defensin 1) salivary level or GLUT2 (glucose transporter 2) and TAS1R2 (Taste receptor type 1, member 2) genotypes are associated with caries risk. The immune system affects oral microbiome through the reaction of the immune cell network at the gingival barrier or by secretory immunity, mainly created by two principal antibody classes present in saliva (secretory IgA (SIgA) and IgG) [93]. Bacterial adhesion and biofilm formation depend on surface properties, such as surface energy, charge, topography, and stiffness of substratum material, which apply to both natural tooth tissues such as enamel, dentin or cement, as well as fillings and dentures [94]. It is well documented that factors such as diet, tobacco use, alcohol, oral hygiene and socioeconomic status cause shifts in the composition of the microbiome, contributing to, among others, the development of caries and periodontal diseases [93].

For example, long-term use of betel quid inhibits the growth of commensal bacteria and induces changes in the abundance of common taxa of oral microbiome. The difference was even more pronounced between betel nut users, regardless of whether they were chewing tobacco, expressed as differences in α and β bacterial diversity. The excessive alcohol consumption can also cause changes in β diversity in the betel-consuming population [95].

Studies showed that current and non-current smokers have different oral microbiota compositions. In both the oro- and nasopharynx, smoking systematically affected the distributions of numerous genera, and there was an enrichment of anaerobic lineages linked with periodontal disease in the oropharynx. Current smokers showed a lower relative abundance of the phylum *Proteobacteria* (4.6 percent) than never-smokers (11.7 percent), with no difference between former and never-smokers. Simultaneously, in current smokers compared with never-smokers, *Capnocytophaga*, *Peptostreptococcus* and *Leptotrichia* had lower abundance, while *Atopobium* and *Streptococcus* were enriched [96,97].

It is worth emphasizing that the composition of the microbiome depends, among other factors, on the study population. The use of the latest metagenomic techniques has allowed identification of thousands of new species of human microbiome in the gut, oral and skin microbiome [35,36,37]. It has been proved that the occurrence of phyla in the gut microbiome is markedly different between urban populations following a high-fat diet and rural populations on a low-fat diet. Westernization is associated with a loss of microbial diversity, including organisms capable of fermenting a diet rich in fibers [38,39].

## 9. Oral Microbiome and Oral Diseases

**Figure 1:**
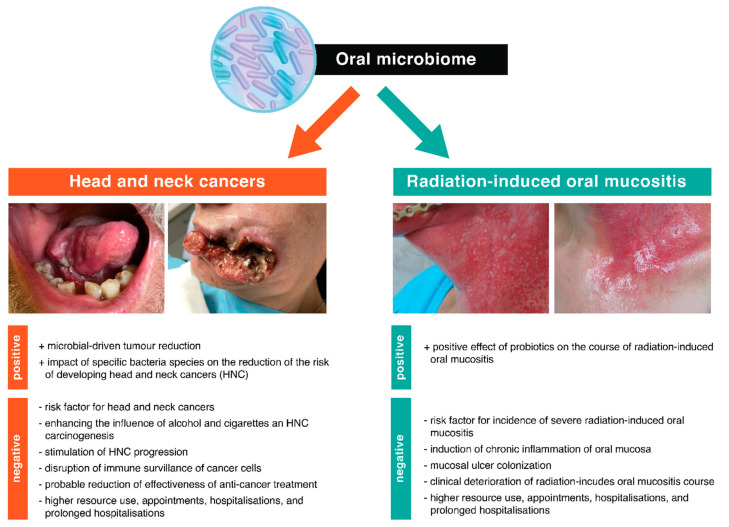
A summary of the effects of the oral microbiome on head and neck cancer and radiation-induce oral mucositis. The figure shows the influence of the oral microbiome on head and neck cancers and radiation-induce oral mucositis, taking into account the positive and negative health effects. A detailed description of the topics discussed in the figure is provided in the manuscript.

The participation of bacteria in the development of diseases such as caries or periodontitis has been clearly documented in numerous studies [98,99,100]. The development of NGS technology and the application of its research into the understanding of the pathogenesis of periodontal diseases allowed for the hypothesis of “key pathogen” [101]. Microbial dysbiosis initiates a multi-stage and complex process that can lead to gingival damage and bone loss [102,103]. This hypothesis assumes that less abundant oral bacteria may act as pathogens under certain conditions, affecting the immune system and thus disrupting the homeostasis of the oral microbiome and, consequently, inducing inflammation. At the same time, they do not directly damage periodontal tissues. The evidence to support the above-mentioned hypothesis is derived from studies into the pathogenesis of periodontitis. It has been shown that certain types of bacteria, such as *Porhyromons gingivalis, Treponema denticola* and *Tannerella forsythia*, despite a slight colonization of the oral cavity surface (less than 0.01% of the total bacterial composition), play a key role in periodontal diseases, disturbing environmental homeostasis. The proposed hypothesis may also be important in explaining the influence of the oral microbiome on the development of RIOM [101]. Figure 1 summarizes the influence of the oral microbiome on head and neck cancers and radiation-induced oral mucositis.

## 10. Oral Microbiome and Chronic Inflammation

One of the best-known examples of how the oral microbiome influences the development of inflammation relates to periodontal disease. Periodontal disease is a bacteria-induced inflammatory condition that causes the loss of the tissues that support the teeth, including the gingiva, as well as the destruction of the alveolar bone. The main factors are *Porphyromonas gingivalis, Treponema denticola and Tannerella forsythia,* which are commensal organisms. Disrupted homeostasis, which include a shift in the oral microbiota, known as dysbiosis, may result in alterations in the relative abundance of individual components of the bacterial community [103]. Neutrophils, normally present in periodontal tissues with sentinel capacity, are key immune cells for initial inflammation and its resolution, and neutrophil abnormalities, including impaired adhesion, cytokine signaling, and phagocytosis, are becoming increasingly recognized as being of importance to chronic inflammatory disease development. Progressive inflammation begins with recognition of periodontal infections by the immune system, which directly leads to a series of successive events, including, among others, immune cell infiltration, such as monocytes and B and T cells, as well as the generation of proinflammatory mediators (e.g., IL-1, IL-6, IL-8 and TNF families or Regulated on Activation, Normal T-cell Expressed and Secreted proteins (RANTES)) and suppression of anti-inflammatory interleukins (e.g., Il-10) [104,105]. During the initial phase of acute stages of periodontal disease, neutrophils infiltrate the lesions. With the transition from acute to chronic inflammation, the cellular composition changes—lesions become infiltrated with mainly monocytic cells. When monocytes become activated macrophages, they differentiate into osteoclasts and produce tissue-damaging proinflammatory cytokines, speeding up bone resorption [106]. In periodontal disease, monocyte chemoattractant protein-1 (MCP-1) is thought to play a key role in the activation and recruitment of inflammatory and immunological cells [104].

Accumulation of activated macrophages at the site of injury is the characteristic feature of chronic inflammatory diseases [107]. In activated neutrophils and macrophages, phagocyte nicotinamide adenine dinucleotide phosphate (NADPH) oxidase is the primary generator of ROS [108]. It is one of the key processes controlling antimicrobial host defense and inflammation by producing excessive oxidative stress. ROS demonstrates a dual nature of influence, both positive and negative to tissue [109]. Due to its capacity to induce irreversible oxidation of biological molecules such as proteins, lipids, and DNA, ROS actively contributes to chronic tissue injury [110]. Oxidative stress can induce cell death through creating an imbalance in antioxidant glutathione equilibrium as well [111]. Releasing chemokines and reactive oxidants by activated macrophages induces cells damage where the most important process is activation of caspases via either an extrinsic pathway (mediated by cell death receptors, including TNF receptor 1 (TNFR1), TNF-related apoptosis-inducing ligand receptor 1 (TRAIL-R1), TRAIL receptor 2 (TRAIL-R2), and Fas receptor) or an intrinsic pathway (by increased mitochondrial outer membrane permeability and release of apoptogenic proteins such as Cytochrome-c (Cyt-c), Smac/Diablo (second mitochondria-derived activator of caspases/Diablo), apoptosis-inducing factor (AIF), and endonuclease G) of cell death [109].

Simultaneously, ROS, through redox signaling pathways, regulates a variety of biological processes important for tissue maintenance, wound healing, infection defense, and inflammatory process regulation. It mediates early transcription-independent wound responses, activation of various growth factor ligands, activates signaling pathways and gene transcription [109].

As oral microbiome affects oral tissues, an effect has also been proved in systemic disease and immune response such as diabetes and cardiovascular disease. The periodontal pathogen *Porphyromonas gingivalis* has been linked to cardiovascular diseases, lung disease, foetal loss, and rheumatoid arthritis, where presumably the driving factor is local and systemic inflammation. Periodontal bacteria also utilize sophisticated immune subversion mechanisms which can undermine the host response and thereby facilitate persistence at extra-oral sites, and include evasion of complement-mediated killing, disarming leukocyte responses, inhibition of lymphocyte activity, and modulation of toll-like receptor (TLR) signaling [112]. The impact of OM has been relatively well researched with regard to atherosclerosis. Pathogens such as *Chlamydia pneumoniae, Helicobacter pylori and Porphyromonas gingivalis* have been identified within human atherosclerotic plaque [113]. Atherosclerosis begins with a dysfunctional endothelium, resulting in the recruitment of a number of immune cells, such as macrophages and T-cells, into the lesion. In addition to the progressive accumulation of inflammatory cells in atherosclerotic lesions, plaques also undergo regressive changes, which alter their size, cellular composition and stability [104].

## 11. Oral Microbiome and HNC

Changes in the human microbiome could determine an oncogenic effect by (i) inducing chronic inflammatory response, (ii) instigating cellular anti-apoptotic signals, (iii) release and activation of carcinogenic factors, (iv) modulation of anticancer immunity, (v) influence on HNC progression and (vi) influence on xenobiotic metabolism [95,114]. 

The oral microbiome is implicated in both the direct effect on the host cell response and the chronic inflammation that may precede oral squamous cell carcinoma (OSCC). Oncobacteria such as *Streptococcus anginosus, Veillonella parvula, Porphyromonas endodontalis*, and *Peptostreptococcus anaerobius* may contribute to the development of OSCC by increasing inflammation via increased expression of inflammatory cytokines such as IL-6, IL-8, TNFα, IFN-γ (interferon gamma), and (granulocyte-macrophage colony-stimulating factor) GM-CSF [115]. Bacterial products such as endotoxins (LPS), enzymes, and metabolic by-products can cause permanent genetic changes in epithelial cells of the host, resulting in epithelial cell proliferation and/or survival.

One of the hypotheses that explains how bacteria contribute to cancerogenesis is that HNC development relates to the activation of procarcinogenic chemicals. In animal models it has been proved that the gut microbiome has a significant influence on the genotoxicity of heterocyclic aromatic amines (HAs). In rats with a natural microbiome, DNA damage produced by the HAs chemical 2-amino-3-methylimidazo[4,5,f]quinoline (IQ) was three to fivefold higher than in germ-free specimens [116]. This effect also depends on the composition of the microbiome—*Bacteroides fragil*is was linked to a distinct increase in mutagenicity in the presence of HAs. On the other hand, *Lactobacilli* species result in a decrease in mutagenicity [117].

The oral microbiome can also modify the effects of alcohol on cancerogenesis. Pure ethanol has been found to have no carcinogenic effects, but one of the metabolites of ethanol—acetaldehyde has been proved to induce mutagenic effects, due to DNA adducts, DNA crosslinking, aneuploidy, or chromosomal aberrations [118]. Streptococcus species, a Gram-positive aerobic species, have been associated with acetaldehyde production. In addition, *Neisseria* species have shown an increased level of alcohol dehydrogenases (ADH) and production of significant amounts of acetaldehyde (ALD) in the presence of ethanol [119,120,121]. Chronic smoking changes the oral microbiota, causing it to create more ethanol-derived ALD. Along with basic risk factors including alcohol and cigarette use, the oral microbiota may operate as a synergistic risk factor [122].

*Streptococcus anginosus*, commonly found in dental plaque, was frequently reported in OSCC and caused DNA damage in oral mucosa due to increased synthesis of nitrous oxide and cyclooxygenase 2 [123].

Metatranscriptomic analysis of the OSCC-associated microbiome has shown the precise actions that these microorganisms engage in, such as increased metal ion transport and nitrous oxide reductase, as well as tryptophanase and protease activity [124]. Increased ion transport around cancer sites is associated with catalyzing radical ions and the promotion of cancer cell growth [125]. Other functional activities of the OSCC-associated microbiome include anaerobic respiration, proteolysis and the response against oxidative stress or radical species damage [126].

As regards *Porphyromonas gingivalis* and *Fusobacterium nucleatum,* it has been proved that bacteria can promote progression of HNSCC by numerous mechanisms.

*Fusobacterium nucleatum,* a known pathogenic oral species, has been reported to stimulate oral cancer progression via direct interaction with oral epithelial cells through Toll-like receptors. Activation of TLR signaling results in increased IL-6 production and activation of signal transducer and activator of transcription 3 (STAT3), which in turn induces important effectors driving OSCC growth and invasiveness (i.e., cyclin D1, matrix metallopeptidase 9, heparanase) [127]. *Fusobacterium nucleatum* promotes cellular invasion and migration by induction the overproduction of MMP-13 (collagenase 3) through upregulation of mitogen-activated protein kinase p38 and Etk/BMX (*e*pithelial and endothelial *t*yrosine *k*inase/Cytoplasmic tyrosine-protein kinase BMX), S6 kinase p70, and RhoA kinase that drive cellular invasion and migration. It has been proved that lipopolysaccharide of *Fusobacterium* *nucleatum,* which contains 2-keto-3-deoxyoctonate and heptose, may inhibit intrinsic apoptotic pathway of oral epithelial cells. FadA adhesion molecule of *Fusobacterium* *nucleatum* binds to E-cadherin and activates b-catenin signaling that regulates cell proliferation and inflammatory responses in oncogenesis [128,129]. Yost et al. [124]. demonstrated that *Fusobacteria* had a higher number of transcripts or active genes in OSCC sites than any other bacteria, followed by *Selenomonas* and *Prevotella*. Simultaneously, it has been proved that species such as *Bacillales*, *Gemella* and *Neisseria* showed increased activity in healthy sites. In the saliva of OSCC patients, higher abundances of *Prevotella melaninogenica*, *Fusobacterium sp., Veillonella parvula, Porphyromonas endodontalis, Prevotella pallens, Dialister, Streptococcus anginosus, Prevotella nigrescens, Campylobacter ureolyticus, Prevotella nanceiensis, Peptostreptococcus anaerobius* and significant elevation of IL-8, IL-6, TNFα, GM-CSF and IFN-γ were observed.

*Porphyromonas gingivalis* disrupts immune surveillance by generating myeloid-derived dendritic suppressor cells (MDDSCs) from monocytes, which inhibit cytotoxic T-Lymphocyte and induce FOXP3 + Tregs (forkhead box P3) through an anti-apoptotic pathway. It has been reported that *Porphyromonas gingivalis* inhibits effector T-cells through inducing regulatory T-cells by induction of expression of B7-H1 and B7-DC receptors on OSCC cells [95,130]. *Porphyromonas gingivalis* induces overexpression of pro-matrix metalloproteinase-9 (pro-MMP-9) through upregulation of ERK1/2-ETS1, p38/HSP27 (Heat shock protein 27), and PAR/NF-kB pathways responsible for the epithelial to mesenchymal transition (EMT) and enhances the production of MMP-1 and MMP-10. Through the possession of fimbriae (FimA adhesin) *Porphyromonas gingivalis* leads to activation and phosphorylation of cyclin-dependent kinases and, as a consequence, reduction of the expression TP53 and induction of cell proliferation [95,129].

Bacteria such as *Bartonella* can also inhibit an apoptosis by induced NF–kB activation in endothelial cells and induce vascular tumor formation [130].

It is worth emphasizing that oral microbiome may reduce risk of HNSCC. *Corynebacterium* and *Kingella* take part in xenobiotic biodegradation and metabolism pathways that are capable of metabolizing several toxicants found in cigarette smoke, such as toluene, nitrotoluene, styrene, chlorocyclohexane and chlorobenzene. Overrepresentation of the commensal bacterial genera, *Corynebacterium* and *Kingella*, showed reduced risk of HNSCC. Other genera such as *Prevotella nanceiensis, Capnocytophaga leadbetteri,* and *Selenomonas sputigena* were inversely related to HNSCC. *Actinomyces oris* and *Veillonella dentocariosa* were associated with a reduced risk of pharynx cancer, whereas *Parvimonas micra* and *Neisseria* sicca were associated with a reduced risk of oral cancer [95,96].

## 12. Human Microbiome and Anticancer Therapy

Incidence and shift in occurrence of pathogenic bacteria, viruses and fungi during hematopoietic stem cell transplantation, chemotherapy or RT have been recently documented. The summary of these studies is presented in Table 7. However, there is no clear data on what changes in the microbiome may be responsible for the development or aggravation of the radiation reaction [131]. Most studies investigate the mechanisms by which individual oral bacterial populations may influence the development of RIOM. It appears that changes in the adhesive properties of various bacterial species, changes in the activity of bacterial colonies or, finally, changes in gene expression and molecular interactions in the bacterial cell may play an important role here [132].

Hou et al. [131] observed the dynamics of changes in the profile of occurring bacteria, which can be classified as both the physiological flora and the pathological flora, during radiotherapy. With an increase in the total dose of ionizing radiation, 20 types of bacteria, including *Pseudomonas, Treponema*, and *Granulicatella*, showed a greater abundance in the oral cavity. An inverse correlation was observed for 10 other types of bacteria, including *Prevotella, Fusobacterium, Leptotrichia, Campylobacter, Peptostreptococcus* and *Atopobium*, the numbers of which decreased significantly with increasing doses of ionizing radiation. However, radiation treatment did not significantly affect the overall richness and evenness of oral microbiome.

Vesty et al. [133] observed a relative stability in the oral microbiome during a course of RT. The microflora was dominated by species such as *Streptococcus, Fusobacterium and Capnocytophaga.* They observed a positive correlation between the presence of bacteria such as *Capnocytophaga leadbetteri, Neisseria mucosa, Olsenella uli, Parviomonas micra* and *Tannerella forsythia* before the start of radiotherapy, and the presence of a radiation reaction with an intensity >G2. Therefore, it was concluded that the oral microbiome before treatment had a greater impact on the risk of developing RIOM than its changes during RT.

Both radiotherapy and chemotherapy have a significant impact on both the oral and gut microbiomes. Chemotherapy-induced oral mucositis has been associated with a marked loss of commensal bacteria such as *Actinomyces, Streptococcus, Granulicatella*, and *Veillonella* and an increased abundance of pathogenic bacteria such as *Enterobacteriaceae*, *Pseudomonas* and *Staphylococcus* species, as well as *Escherichia coli* or *Fusobacterium nucleatum*. Ulcerative oral mucositis was related to *Porphyromonas gingivalis, Candida glabrata,* and *Candida kefyr* [132,134,135].

Shifts in oral microbiome during anticancer treatment may promote the dominance of mucolytic bacteria such as the *Streptococcus* species which degrade the mucous layer and leads to further oral mucositis development [132,134,135].

It is worth mentioning that the use of probiotics has shown a positive effect on the course of RIOM. In randomized studies of 188 HNC patients undergoing CRT, lozenges containing *Lactobacillus brevis CD2* or a placebo were given once a day. In the study group, a significantly lower incidence of treatment complications was observed compared to the placebo group (52% compared to 77%) [136]. Probiotics compete with oral microorganisms for nutrients by producing bacteriocins capable of inhibiting the growth of pathogens, modulating proliferation/cell apoptosis and stimulation of the mucosal immune system [137]. Similar observations were made in a study on the use of prebiotics. Prebiotics stimulate the growth of beneficial microorganisms and inhibit the activity of pathogenic bacteria. A randomized study of 99 patients undergoing CRT showed that the use of prebiotics reduced the incidence of serious complications compared to the placebo group (15.5% versus 45.7%) [137]. This proves that the change in the composition of the oral microflora influences the response of proliferating tissues resulting from anticancer treatment. Table 8 summarizes the studies examining impact of probiotics on cancer therapy-induced oral mucositis.

Significantly, the intestinal microflora, through its metabolic and immunomodulating activities, can affect the effectiveness of immunotherapy. There is reliable evidence for the effects of the gut microbiome on efficacy of cancer chemotherapy and immunotherapy. Animal studies show that specific composition of the gut microbiome could lead to an increased immunotherapy success rate [86]. Disruption of the microbiota impairs the response of subcutaneous tumors to CpG-oligonucleotide immunotherapy and platinum-based chemotherapy. Both treatments were shown to be less effective in tumor-bearing animals lacking microbiota, which is crucial for triggering the innate immune response against malignancies. A proposed cause for this phenomenon is lower cytokine production and tumor necrosis after CpG-oligonucleotide treatment and deficient production of reactive oxygen species and cytotoxicity after chemotherapy. The gut microbiome primes tumor-infiltrating myeloid cells in a TLR4-dependent manner for enhancing production of ROS upon oxaliplatin treatment, leading to tumor regression [138]. Recent works suggest the potential involvement of the gut microbiome in influencing the efficacy of checkpoint inhibitor treatment strategies for both CTLA-4 (cytotoxic T cell antigen 4) and PD1-targeting checkpoint inhibitors. Moreover, recent research suggests that the microbiome may also have a detrimental effect on anticancer medication stability and half-life in some circumstances, by altering or degrading chemotherapy drugs [139].

Just as the microbiome can influence the response to treatment, recent research suggests the possible utilization of the tumor microbiome in anticancer treatment. *Escherichia coli* and other facultative anaerobic bacteria can infiltrate solid tumors, causing tumor growth retardation or even elimination. The mechanism of microbial-driven tumor reduction is thought to involve the induction of anticancer immune responses, such as bacteraemia-induced TNF secretion, as well as expression of granzyme B, FasL, TNFα and IFN-gamma resulting in CD8+ T cell activation, which aids tumor surveillance and clearance [140].

## 13. Future Perspective

Numerous studies indicate the presence and development of pathogenic bacteria in the oral cavity environment at various stages of anticancer treatment. However, there is no clear data on what changes in the microbiome may be responsible for the development or exacerbation of the radiation reaction. Awareness of risk factors for the development of RIOM dependent on the microbiome is necessary in light of the fact that intensified adverse reactions may cause the need to halt RT, and thus reduce its effectiveness. Therefore, it is important to understand the cause of these complications in order to introduce effective, targeted treatment [141]. Obtaining new information on the composition of the oral microbiome and the dynamics of its changes during and after treatment will contribute to the broadening of knowledge of the biological role of commensal and pathological oral microflora in the process of RIOM formation. Additionally, it may also become the basis for further analysis of the emerging late radiation reactions.

## 14. Conclusions

Head and neck cancer is a multifactorial and complex disease. The incidence rates and location of HNC vary worldwide mainly due to different lifestyles and associated risk factors. Numerous studies have indicated bacterial influence on oral diseases, the formation of chronic systemic inflammation and potential to influence carcinogenesis. A 16S rRNA metagenomics with NGS has contributed significantly to a better understanding of the oral microbiome. Understanding how the characteristic composition of the oral microflora influences the emerging acute radiation reaction may allow for the development of a multifactorial model to predict the development and severity of RIOM, and the optimization of personalized prevention and treatment of the resulting inflammatory radiation complications aimed at the mechanism of their formation.

There is sufficient evidence in the literature to confirm the influence of the oral microbiome on the development of both systemic diseases and the development of neoplasms. Though normal oral bacterial flora may not have a direct role in epithelial dysplasia and OSCC, they may be of significance when they occur in combination with other established etiological variables such as smoking and alcohol consumption. Furthermore, clinicians need to be aware of the influence of the microbiome on the effectiveness of anticancer treatment and the development of side effects.

Further studies are needed to better establish the structure of the oral microbiome as well as the dynamics of its changes before and after therapy. It will help to expand the understanding of the biological function of commensal and pathogenic oral microbiota in the HNC carcinogenesis and development of radiation-induced oral mucositis.

## Figures and Tables

**Table 1:** The impact of regular dental visits on the risk of developing head and neck squamous cell carcinoma.

References	Year	Rated Factor	Type of Study	*n*	Risk of HNC	Description
Lissowaska et al. [29].	2003	Lack of dental care	case–control study	122 cases 124 controls	Increased (in the group with no dental care)	OR = 11.89 95% CI: 3.33–42.51
Rosenquist et al. [30].	2005	Regular dental care	case–control study	132 cases 320 controls	Decreased (in the group with regular dental care)	OR = 0.4 95% CI: 0.2–0.6
Guha N et al. [31].	2007	Lack of dental care	case-control study	2286 cases 1824 controls	Increased (in the group with no dental care)	OR = 1.61 95% CI: 1.18–2.20
Divaris et al. [32].	2010	Regular dental care	case-control study	1361 cases 1289 controls	Decreased (in the group with regular dental care)	OR = 0.68 95% CI: 0.53–0.87
Chang JS, et al. [33].	2013	Lack of dental care	case-control study	317 cases 296 controls	Increased (in the group with no dental care)	OR = 2.86 95% CI: 1.47–5.57
Ahrens et al. [34].	2014	Lack of dental care	case-control study	1963 cases 1993 controls	Increased (in the group with no dental care)	OR = 1.93 95% CI: 1.48–2.51
Hashim et al. [35].	2016	Regular dental care	case-control study	8925 cases 12,527 controls	Decreased (in the group with regular dental care)	OR = 0.78 95% CI: 0.72–0.85

OR—odds ratio, CI—confidence intervals; HNC—head and neck squamous cell carcinoma.

**Table 2:** The impact of occurrence of missing teeth on risk of developing head and neck squamous cell carcinoma.

References	Year	Rated Factor	Type of Study	*n*	Risk of HNC	Description
Pereira et al. [36].	2020	missing teeth (6 or more)	case-control study	899 cases 899 controls	Increased (in the group with missing teeth)	OR = 3.30; 95% CI: 2.67—4.08
Gupta et al. [37].	2020	missing teeth (5 or more)	case-control study	240 cases 240 controls	Increased (in the group with missing teeth)	OR = 3.24; 95% CI: 2.09—5.01
Kawakita et al. [38].	2017	missing teeth (5 or more)	case-control study	484 cases 313 controls	Increased (in the group with missing teeth)	OR = 2.68; 95% CI: 2.09—3.43
Liu et al. [39].	2016	missing teeth a—1—3 missing teeth b—4—13 missing teeth c—more than 14	case-control study	2528 cases 2596 controls	Statistically insignificant *	a—OR = 0.97; 95% CI: 0.79, 1.19 b—OR = 0.98; 95% CI: 0.76, 1.28 c—OR = 0.71; 95% CI: 0.46, 1.11
Chang et al. [33].	2013	missing teeth a—1—10 missing teeth b—11—20 missing teeth c—more than 20	case-control study	317 cases 296 controls	Increased (in the group with missing teeth)	a— OR = 1.15; 95% CI: 0.61–2.20 b— OR = 1.34; 95% CI: 0.58–3.07 c— OR = 2.40; 95% CI: 0.97–5.97

OR—odds ratio, CI—confidence intervals; HNC—head and neck squamous cell carcinoma; * the study concerned only nasopharyngeal neoplasms.

**Table 3:** The impact of occurrence of periodontal disease on risk of developing head and neck squamous cell carcinoma.

References	Year	Rated Factor	Type of Study	*n*	Risk of HNC	Description
Gupta et al. [37].	2020	Periodontitis a—mild b—moderate c—severe	case-control study	212 cases 188 controls	Increased (in the group with periodontitis)	a— OR = 1.92; 95% CI: 0.93—3.96 b— OR = 2.47; 95% CI: 1.29—4.72 c— OR = 2.75; 95% CI: 1.45—5.23
Pereira et al. [36].	2020	Periodontitis (expressed as gingival bleeding)	case-control study	899 cases 899 controls	Increased (in the group with periodontitis)	OR = 2.40 ; 95% CI: 1.40—4.09
Shin et al. [40].	2019	Periodontitis a—incipient b—severe	case-control study	146 cases 278 controls	Increased (in the group with periodontitis)	a—OR = 3.463; 95% CI: 1.348—8.895 b—OR = 4.066; 95% CI: 1.499 –11.026
Khan et al. [41].	2019	Periodontitis	case-control study	276 cases 275 controls	Increased (in the group with periodontitis)	OR = 5.04; 95% CI: 3.18—8.01
Moergel et al. [42].	2013	Periodontitis (expressed as mean bone loss)	case-control study	178 cases 123 controls	Increased (in the group with periodontitis)	OR = 2.4; 95% CI: 1.5—3.8
Zeng XT et al.,[43].	2013	Presence of periodontal disease	meta-analysis		Increased (in the group with periodontitis)	OR = 2.63 95% CI: 1.68–4.14

OR—odds ratio, CI—confidence intervals; HNC—head and neck squamous cell carcinoma.

**Table 4:** The impact of oral hygiene habits on risk of developing head and neck squamous cell carcinoma.

References	Year	Rated Factor	Type of Study	*n*	Risk of HNC	Description
Pereira et al. [36].	2020	flossing (flossing regularly vs. flossing sometimes)	case-control study	899 cases 899 controls	Decreased (in the group flossing regularly)	OR = 0.16; 95% CI: 0.08—0.33
Gupta et al. [37].	2020	Tooth brushing (<2 times/day vs ≥2 times/day)	case-control study	212 cases 188 controls	Increased (in the group brushing teeth <2 times/day)	OR = 2.09 95% CI: 1.27—3.45)
Kawakita et al. [38].	2017	Tooth brushing (<2 times/day)	case-control study	484 cases 313 controls	Increased	OR = 1.77; 95% CI: 1.46—2.15
Chen et al. [44].	2016	Tooth brushing (≥2 times/day vs <2 times/day)	case-control study	250 cases 996 controls	Decreased (in the group brushing teeth ≥2 times/day)	OR = 0.50; 95% CI: 0.25—0.98
Hashim et al. [35].	2016	Tooth brushing (≥1/day vs. <1/day)	case-control study	7 411 cases 10 333 controls	Decreased (in the group brushing teeth ≥1 times/day)	OR = 0.83; 95% CI: 0.68— 1.00
Tsai et al. [45].	2014	Tooth brushing (<2 times/day vs ≥2 times/day)	case-control study	436 cases 514 controls	Increased (in the group brushing teeth <2 times/day)	OR = 1.40; 95% CI: 1.02—1.91
Chang et al. [33].	2013	Tooth brushing (<2 times/day vs ≥2 times/day)	case-control study	317 cases 296 controls	Increased (in the group brushing teeth <2 times/day)	OR = 1.5; 95% CI: 1.02—2.23
Sato et al. [46].	2011	Tooth brushing (never vs. ≥2 times/day)	case-control study	469 cases 2696 controls	Increased (in the group never brushing teeth)	OR = 2.86; 95% CI: 1.07—7.66
Wu et al. [47].	2017	Tooth brushing (<2 times/day)	case-control study	242 cases 856 controls	Increased	OR = 1.50 95% CI: 1.08—2.09

OR—odds ratio, CI—confidence intervals; HNC—head and neck squamous cell carcinoma.

**Table 5:** Biological pathways involved in radiation-induced oral mucositis.

Nitrogen metabolism
Toll-like receptor signaling
Nuclear Factor—kappa B (NF-κB) signaling
B Cell receptor signaling
PI3K-AKT signaling
Cell Cycle: G2/M DNA damage checkpoint receptor
P38 MAPK signaling
Wnt/B-catenin signaling
Glutamate receptor signaling
Integrin signaling
VEGF signaling
IL-6 signaling
Death receptor signaling
SAPK/JNK signaling

PI3K—phosphatidyl inositol 3-kinase; AKT-v-akt murine thymoma viral oncogene homolog 1; NF-κB—nuclear factor—kappa B; p38 MAPK—p38 mitogen-activated protein kinases; VEGF—vascular endothelial growth factor; IL-6—interleukin 6; SAPK—stress-activated protein kinases; JNK—Jun amino-terminal kinases.

**Table 6:** Comparison of description of radiation-induced oral mucositis scales.

Scale	Grade 0	Grade 1	Grade 2	Grade 3	Grade 4	Grade 5
WHO [78].	No change	Oral soreness/erythema	Erythema, ulcers, can eat solids	Ulcers; requires liquid diet only	Alimentation not possible	N/A
EORTC/RTOG [79].	No change over baseline	mild pain, not requiring analgesic	Patchy mucositis, serosanguinous discharge. May experience pain requiring analgesics, lesion <1.5 cm, noncontiguous	Confluent fibrinous mucositis/may include severe pain requiring narcotics, lesion > 1.5 cm, contiguous	Necrosis or deep ulceration, ±bleeding	Death
CTCAE 5.0 [80].	N/A	Asymptomatic or mild symptoms; intervention not indicated	Moderate pain or ulcer that does not interfere with oral intake; modified diet indicated	Severe pain; interfering with oral intake	Life-threatening consequences; urgent intervention indicated	Death
OMAS [81].	Lesions = none erythema = none	Lesions = < 1 cm^2^ erythema = not severe	Lesions = 1–3 cm^2^ erythema = severe	Lesions = > 3 cm^2^	N/A	N/A
OMI [82].	Assesses clinically evident oral mucosal changes (atrophy, erythema, ulceration, pseudomembranous ulcerations, and edematous changes) and consists of 34 items, each scaled from 0 to 3 (normal to severe).					
WCCNR [83].	Lesions = none erythema = 50% or more pink bleeding = none	Lesions = none erythema = 50% or more slightly red bleeding = none	Lesions = none erythema = 50% or more moderately red bleeding = with eating or mouth care	Lesions = none erythema = 50% or more very red bleeding = spontaneous	N/A	N/A
SWOG[84].	None	Painless ulcers, erythema or mild soreness	Painful erythema, oedema, or ulcers, but can eat	Painful erythema, oedema, or ulcers, and cannot eat	Requires parenteral or enteral support	-

WHO—World Health Organisation Oral Toxicity Scale, RTOG/EORTC—Radiation Therapy Oncology Group/European Organisation for Research and Treatment of Cancer, CTCAE—Common Terminology Criteria for Adverse Events; OMAS—Oral mucositis Assessment Score; OMI—Oral Mucositis Index; WCCNR—Western Consortium for Cancer Nursing Research Scale; SWOG—Southwest Oncology Group.

**Table 7:** Synthesis of studies investigating microbiome during radio- or chemoradiotherapy in patients with head and neck cancer.

References	Cancer	Treatment	Number of Patients; *n*	Method	Time Point for Measurements	Conclusion	Full Mouth Clinical Examination	Materials
Hou et al. [48].	HNC	RT	19	16S rRNA, V4 gene	8 points: before RT, 10, 20, 30, 40, 50, 60, 70 Gy	20 genera—significantly positively associated with their radiation dose; 10 genera—negatively associated	+	swabs
Vesty et al. [50].	HNC	RT/CRT	19	16S rRNA, V3-V4 gene ITS1/ITS2 for fungi	3 points: 0–20; 21–40; 41–60 Gy	microbiota remain stable during RT; periopathogenic genera *Porphyromonas* and *Tannerella*, were all positively correlated with ≥ grade 2 OM	−	Saliva, swabs
Zhu et al. [58].	HNC	RT/CRT	19	16S rRNA, V4 gene	8 points: before RT, 10, 20, 30, 40, 50, 60, 70 Gy	1. increase in the relative abundance of some Gram-negative bacteria; 2. severe mucositis harbored a significantly lower bacterial alpha diversity and higher abundance of *Actinobacillus*	+	swabs
Reyes-Gibby et al. [59].	HNC	RT/CH/CRT	66	16S rRNA, V4 gene	8 points	Changes in the abundance of genera over the course of treatment were associated with the onset of severe OM.	−	swabs
Hu et al. [60].	HNC	RT	8	16S rRNA, V1-V3 gene	2 points: before and after RT	Temporal variation of major cores in relative abundance, negative correlation between the number of OTUs and radiation dose	+	Supragingival plaque
Hu et al. [61].	HNC	RT	8	16S rRNA, V1-V3 gene	7 points: once per week	Fluctuations in gen era synergistically involved in the development of RIOM	+	Supragingival plaque

HNC—head and neck cancer; RT—radiotherapy; CT—chemotherapy; CRT—chemoradiotherapy, rRNA—ribosomal ribonucleic acid; OM—oral mucositis; OTU—Operational taxonomic unit; RIOM– radiation-induced oral mucositis.

**Table 8:** Clinical trials on prevention and treatment of cancer therapy-induced oral mucositis with probiotics.

References	Cancer	Treatment	Intervention	Type of Study	Number of Patients; *n*	Conclusion
Limaye et al. [137].	HNC	CT	AG013 *Lactococcus lactis *vs. placebo ratio 5:1	RCT Single-blinded	52	35% decrease in mean percentage of days with ulcerative oral mucositis as compared to placebo
Sharma et al. [136].	HNC	CRT	*Lactobacillus brevis* CD2 vs. placebo ratio 1:1	RCT Double-blinded	210 efficacy analysis—188	Decrease incidence of 3–4 grade oral mucositis (52% vs 77%; *p* < 0.001);
Sanctis et al. [142].	HNC	CRT	*Lactobacillus brevis* CD2 vs. bicarbonate mouthwash	RCT open-label	75 efficacy analysis—68	No statistical difference in the incidence of grade 3–4 oropharyngeal mucositis between the intervention and control groups (40.6% vs. 41.6% respectively, *p* = 0.974)
Jiang et al. [143].	HNC	CRT	Probiotic combination *(Bifidobacterium longum, Lactobacillus lactis, and Enterococcus faecium)* vs. placebo ratio 2:1	RCT Double-blinded	99	Significant reduction in the severity of OM (grade 3—15.52% vs 45.71%; *p* < 0.001)

HNC—head and neck cancer; RT—radiotherapy; CT—chemotherapy; CRT—chemoradiotherapy, RCT—randomised clinical trials.
